# Fate and freedom in developing neocortical circuits

**DOI:** 10.1038/ncomms16042

**Published:** 2017-07-03

**Authors:** Denis Jabaudon

**Affiliations:** 1 Department of Basic Neurosciences, Geneva University, 1 rue Michel Servet, 1211 Geneva, Switzerland; 2 Clinic of Neurology, Geneva University Hospital, 1 rue Michel Servet, 1211 Geneva, Switzerland; 3 Geneva Neurocenter, Geneva University, 1 rue Michel Servet, 1211 Geneva, Switzerland

## Abstract

The activity of neuronal circuits of the neocortex underlies our ability to perceive the world and interact with our environment. During development, these circuits emerge from dynamic interactions between cell-intrinsic, genetically determined programs and input/activity-dependent signals, which together shape these circuits into adulthood. Building on a large body of experimental work, several recent technological developments now allow us to interrogate these nature–nurture interactions with single gene/single input/single-cell resolution. Focusing on excitatory glutamatergic neurons, this review discusses the genetic and input-dependent mechanisms controlling how individual cortical neurons differentiate into specialized cells to assemble into stereotypical local circuits within global, large-scale networks.

## Abstract (web summary)

Proper functioning of the neocortex – the center of higher-order brain functions – depends on the correct assembly of neocortical neural circuits during development. Here the author discusses how cell-intrinsic developmental programs and activity-dependent signals together shape the formation of neocortical circuits.

The neuronal circuits of the neocortex are at the root of our idiosyncratic ability to perceive the world and conduct meaningful interactions with our surroundings. Neocortical circuits, through their activity, encode for critical behaviorally relevant processes, including sensory perception and integration, sensory-motor transformation, motor planning and execution, and long-term memory and attention. These circuits are formed by a diversity of specialized subtypes of neurons, which can be distinguished from each other by anatomical, morphological, physiological, hodological (that is, relating to connectivity) and genetic features. During development, these neurons assemble into serial and parallel arrays of hierarchically interlinked circuits that exhibit specialized and stereotypical organization and together account for cortical function.

The neocortex is radially organized into layers, which each are enriched in specialized subtypes of neurons, and tangentially organized into areas, which specialize in distinct sensory, motor, and associative functions (Fig. 1a). Neocortical circuits are both robust and flexible, reliably carrying out complex multimodal tasks while also being able to modify the execution of these tasks in response to context and previous experience. To accommodate the seemingly opposing constraints of reliability and plasticity, at least two main driving forces have emerged during evolution and are at play during development: (1) cell-autonomous, genetically determined processes, which generate a core set of specialized neuronal subtypes and (2) non cell-autonomous, input/activity-dependent processes, which act during critical periods of development to refine these neurons into further subtypes, enabling neural circuit diversification and context-dependent expansion of the behavioural repertoire. A proper balance between these intrinsic developmental programs and external signals is essential for the differentiation and assembly of neurons into circuits, yet the actual contribution of these two types of processes to cortical development remains unclear.

Until recently, only relatively low-resolution tools were available to study the interaction between external signals and intrinsic developmental transcriptional programs. This is true both for genetic interventions, in which targeted disruption of gene expression was a cumbersome and often unreliable task (and also was constitutive across tissues), as well as for the limited ability to manipulate input without resorting to lesions of peripheral sensory organs. Directed genetic manipulations, for example using CRISPR/CAS9-type approaches, single-cell RNA sequencing, and chemogenetic and optogenetic manipulations of single-neuron types *in vivo* now allow cell-type-specific manipulation of gene expression and activity, from the earliest stages of embryogenesis until adulthood. Altogether, these tools are ushering in a new era in the study of how interactions between genes and environment shape developing cortical circuits in health and disease.

The aim of this review is to provide an integrated overview of molecular and input/activity-dependent parameters that define the configuration of neocortical circuits. Focusing on excitatory glutamatergic neurons, I will first discuss the laminar and areal organization of the distinct specialized neocortical neuron subtypes, and their intracortical connectivity and reciprocal interactions with neurons from other brain regions, including the thalamus. I will then discuss the cell-intrinsic and -extrinsic mechanisms controlling the developmental emergence of these neurons and their ability to self-organize into circuits, and conclude with a discussion of unresolved questions and disease/repair-related perspectives.

## Functional organization of neocortical circuits

### Laminar organization of the neocortex

**Figure 1:**
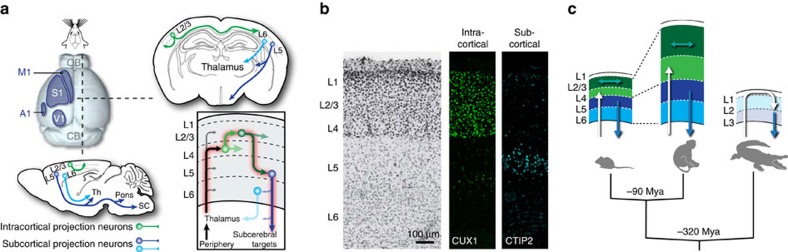
Areal and laminar organization of the neocortex. (**a**) Schematic representation of the distinct primary cortical areas in the mouse, and cell-type specific connectivity of glutamatergic cortical neurons. A1: primary auditory cortex, M1 primary motor cortex, S1: primary somatosensory cortex, V1: primary visual cortex. (**b**) Laminar organization of the neocortex (S1). CUX1 specifically labels intracortical projection neurons while CTIP2 labels corticospinal neurons in layer (L) 5. (**c**) Laminar organization of the neocortex in mammals and reptiles. Mya: millions of year ago.

The neocortex is anatomically composed of six layers of cells, corresponding to distinct densities of neuronal somas, dendrites and axons. These laminae are not only anatomical landmarks, but consist of developmentally and functionally distinct subtypes of glutamatergic neurons (Fig. 1a,b)^1^,^2^. The deepest cortical layers contain neurons whose axons target subcortical structures such as the thalamus (corticothalamic neurons, layer (L6) and the tectum, hindbrain and spinal cord (‘corticospinal’ neurons, L5). In contrast, neurons located more superficially in layers 2 and 3 (L2/3) and 4 (L4) have intracortical axonal targets. Neurons in L4 (also called the ‘granular layer’) differ from the pyramidal projection neurons present in other layers in that they are locally projecting glutamatergic interneurons. They are the main targets of neurons in exteroceptive sensory thalamic nuclei (that is, which receive input from the sense organs)^3^,^4^,^5^ and, as such, form the principal sensory gateway to the neocortex. L4 neurons appear to be particularly sensitive to impairments of the sensory organs or their input pathways. As will be discussed in detail later, this is particularly striking in the rodent somatosensory cortex, where whisker-input receiving L4 neurons are clustered into distinct cellular assemblies called ‘barrels’^4^,^5^.

Infragranular (that is, L5/6) and supragranular (L2/3) layers have different evolutionary histories since the latter layers are only found in extant mammals^6^ (Fig. 1c). The dramatic expansion of intracortically projecting neurons within superficial layers is thus an evolutionarily novel feature of mammals, with no equivalent in other vertebrates. This addition of cortical layers may have been enabled by a shift in the position of thalamocortical axons, which reach the cortex from above in reptiles but from below it in mammals^7^. Of note, intracortical projection neurons are also found in deep cortical layers, within which some of these neurons also have projections to the striatum^8^. These infragranular intracortical projection neurons may correspond to ‘ancestral’ forms of intracortically projecting neurons, which also likely exist in reptiles. Thus, hundreds of millions of year of convergent evolution may separate infragranular and supragranular intracortically projection neurons. Whether L4-type neurons are already present in reptiles is unclear, although recent evidence indicates that a layer of cells expressing the L4 marker RORB is present in the dorsal cortex of turtles^9^.

Excitatory neocortical neurons are born successively during corticogenesis, which extends between embryonic days 10.5 and 18.5 in the mouse, and between postconceptual week 7–27 in humans^10^. Neurons located in the deepest cortical layers are born first, while neurons located more superficially are born later (see Generation of specialized neocortical cell types, below). Since they are generated later, neurons located in superficial layers develop at a time, when sense organs may be conveying signals generated by interactions with the environment. In fact, L2/3 neurons are still migrating during the first postnatal days in mouse, a time when neurons in infragranular layers are already over a week old. The coexistence of neurons at different stages of maturation during circuit assembly implies that the environment could have different effects on distinct cell types during ‘critical periods’ of development, relating to the time of birth of target cells. Supporting this possibility, in the somatosensory cortex, synaptic plasticity decreases postnatally in a sequence which corresponds to neuronal date of birth^11^,^12^. Similarly, within single neurons, early occuring events are less sensitive to input than later-occurring ones. For example, laminar positioning, which occurs early, is a largely fixed feature, while neuritogenesis, which occurs at later developmental stages, remains plastic into adulthood^13^. The coincident timing of molecular processes in neurons born at the same time across cortical areas/brain regions could thus provide a parsimonious mechanism for circuit assembly during corticogenesis. However, it is for instance not sufficient to explain the coordinated assembly of excitatory and inhibitory neurons^14^. Thus, whether temporal relationship between cell type is instructive for their circuit assembly remains unclear.

In addition to glutamatergic neurons, whose diversity and circuit identity are the focus of the current review, the neocortex contains another population of neurons, which are GABAergic and are born from distinct germinal zones than excitatory neurons (see Developmental emergence and plasticity of neocortical circuits). Distinct subtypes of GABAergic neurons migrate into the cortex during late corticogenesis in mouse and integrate circuits within which they act as inhibitory interneurons. These interneurons play a pivotal role in the gating and spread of cortical signals through processes such as feedforward inhibition, dis-inhibition and feedback inhibition. This diverse neuronal population, and its role in cortical information processing has been described in a recent review^15^, and they will not be covered in detail here.

### Area-specific interactions with the thalamus

Layers are not homogenous across the rostro-caudal and latero-medial extent of the neocortex. Instead, local cytoarchitecture varies in a sometimes discontinuous way across the tangential surface of the brain, defining distinct cortical areas. Histological discontinuities are particularly striking in species with large cortices, and form the basis of the Brodmann classification of cortical areas^16^, in which cytoarchiturally defined areas have specific functional roles within motor and sensory hierarchies.

**Figure 2:**
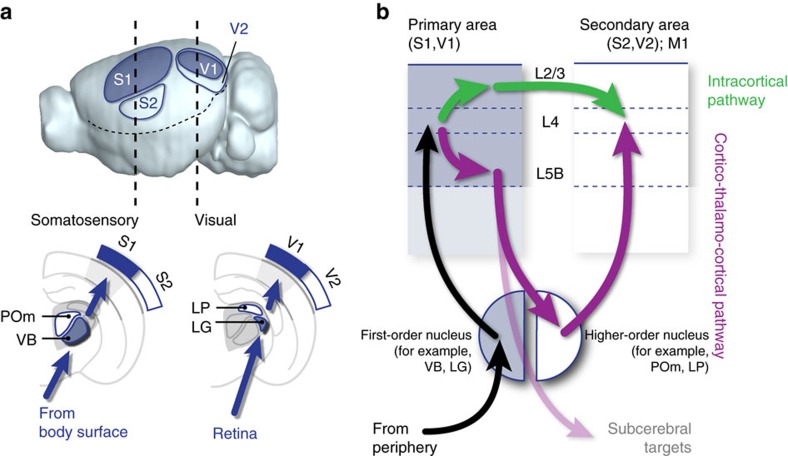
Cortical information flow. (**a**) Exteroceptive, first-order thalamic nuclei (filled in blue) project to primary cortical areas (e.g.S1, V1). Higher-order thalamic nuclei and secondary cortical areas are outlined in blue. POm: posteromedial thalamic nucleus; LG: dorsolateral geniculate nucleus; LP: lateroposterior nucleus; VB: ventrobasalis nucleus. (**b**) Two main pathways allow inter-areal communications: an intracortical pathway (green) and a cortico-thalamo-cortical pathway (purple), originating from L5B corticospinal neurons and which transits through higher-order thalamic nuclei.

Each cortical area is reciprocally connected with a defined subset of thalamic inputs (Fig. 2a). Frontally located cortical areas are connected with rostrally located thalamic nuclei, including those involved in motor planning and execution, while parieto-occipital and temporal cortical areas are interconnected with more posterior thalamic nuclei, and are involved in sensory perception and integration^17^,^18^. Delineations between individual cortical areas are particularly sharp in areas receiving input from exteroceptive thalamic nuclei, such as the primary somatosensory, visual and auditory cortices (Brodmann areas 1–3, 17 and 41–42, respectively). Because of their characteristic cytoarchitectural features, primary sensory areas, and particularly the primary somatosensory (S1) and visual (V1) areas, have been extensively used as model systems to study the role of input on cortical differentiation. As a consequence, our understanding of thalamocortical organization and information flow is largely based on the connectivity of primary sensory areas, and particularly somatosensory and visual areas, since the functional connectivity of the primary auditory area (A1) is much less well understood^19^,^20^. However, these areas only represent a small fraction of the total cortical surface, and different connectivities and information flow exist in other cortical areas and thalamic nuclei, as will be detailed in the next section.

### Cortical information flow

Sensation starts with detection of a stimulus through activation of peripheral receptors, such as skin mechanoreceptors or retinal photoreceptors. Input from these receptors reaches neurons located in exteroceptive, ‘first-order’ thalamic nuclei such as the ventrobasalis nucleus (VB, for tactile stimuli), the dorsolateral geniculate nucleus (LG, for visual stimuli) and the ventral medial geniculate nucleus (vMG, for auditory stimuli). Sensory information then reaches primary sensory areas of the neocortex, where core stimulus properties are perceived, and is then forwarded to secondary sensory (for example, S2 and V2) and associational areas where stimulus features are dynamically and multi-modally processed (Fig. 2a).

Within primary sensory areas, first-order nuclei project particularly strongly onto L4 neurons, which act as the main entry point of extracortical input^5^. From L4 neurons, information is then split into two parallel streams (Fig. 2b): first, an intracortical stream, through which signals are sent to distinct subtypes of L2/3 intracortical neurons, which project to specific sets of cortical areas, including S2, V2, and M1; second, a cortico-thalamo-cortical stream, in which L4 signals are sent to infragranular L5B neurons, which send top-down projections to non-exteroceptive, ‘higher-order’ thalamic nuclei (posteromedial nucleus (POm), from S1; lateroposterior nucleus or pulvinar (LP) from V1), and dorsal medial geniculate nucleus (dMG), from A1). Higher-order thalamic nuclei in turn project to L4 neurons of secondary sensory (that is, S2, V2) areas, thus closing a cortico-thalamo-cortical loop^21^,^22^. Higher-order thalamic nuclei do not project exclusively to secondary sensory areas, but instead have diffuse connections across many cortical areas^18^. As such, they may be involved in the coordination of activity across motor and sensory cortices during active sensing, as occurs in the somatosensory system when mice sweep their whiskers back and forth to generate a tactile representation of their environment.

In addition to these two main information streams, multiple entry points exist into primary cortices. These include direct projections from first-order thalamic afferents onto L5B and L6 neurons, and projections from higher-order nuclei to L5A intracortical neurons^3^. Similarly, additional output points from primary cortices include L6 corticothalamic neurons, which project to corresponding sensory thalamic nuclei^18^,^23^.

These two main inter-areal communication pathways have distinct evolutionary histories, since supragranular intracortical projection neurons are a novel acquisition of mammals. In the absence of such intracortical projections, diffuse cortico-thalamo-cortical circuits may have been the main pathway allowing different cortical regions to communicate with one another, as might still be the case in reptiles. By providing a novel pathway to direct information to specific brain areas, supragranular intracortical projection neurons in mammals have thus potentially allowed the untethering of cortical function from input–output thalamocortical loops, and emergence of stimulus-dissociated, integrative neocortical functions.

### Linking sensory and motor areas

The diversity of intracortical projection neurons and the mechanisms controlling their areal target specificity are still poorly understood, and have mostly been studied for S1 to primary motor cortex (M1) connections. Recent work has shown that distinct subtypes of neurons project from S1 to M1 or from S1 to S2 (refs 24, 25), suggesting a high degree of specialization in intracortical projection neurons. However, whether similar neuronal specialization exists for other cortical targets or is also present in other primary cortical areas is still unclear. S1 and M1 are hierarchically linked in that information flows from S1 to M1; in this respect, M1 can be seen as a higher-order somatosensory region. The exact function of M1 and the nature of the circuits of this area are, surprisingly, still not well understood. For example, activation of S1 L5B corticospinal neurons is sufficient to induce muscle contraction^26^ such that motor action does not necessarily require M1 activity.

It is difficult to establish a strict first- and higher-order classification for thalamic input to motor cortical areas. While L4 neurons are not visible as such in M1, cells with L4-type circuit connectivity have been identified^27^, and the ventro-anterior (VA) and ventrolateral (VL) thalamic nuclei have M1 connectivities reminiscent of first- and higher-order nuclei, respectively^28^,^29^. Therefore, canonical intracortical and cortico-thalamo-cortical circuits may be present across cortical areas. Thus, cortical circuits emerge from the combinatorial assembly of spatially, evolutionarily and developmentally distinct subtypes of neurons. In the next section, I will discuss the developmental controls over the emergence of these distinct specialized neurons during corticogenesis.

## Developmental emergence and plasticity of neocortical circuits

The processes allowing the emergence and functional specialization of cortical circuits start with the generation of neurons from progenitors and extend into adulthood, through experience-dependent developmental processes. In this section, I will first discuss the developmental diversity of neocortical progenitors and excitatory neurons. Next, I will present select cell-intrinsic mechanisms controlling the type-specific differentiation of these neurons. Finally, I will discuss some of the input-dependent processes that shape cortical circuits into area-specific functional networks.

### Generation of specialized neocortical cell types

The neurons that form the distinct layers of the neocortex are sequentially born within two main germinal zones between E10.5 and E18.5 in the mouse: the ventricular zone (VZ) of the dorsal pallium, which gives rise to excitatory glutamatergic neurons^1^, and a seemingly heterogeneous ventral pallial VZ, including the medial and caudal ganglionic eminences and pre-optic area, which gives rise to distinct subtypes of cortical inhibitory GABAergic interneurons. As mentioned earlier, the latter cell type will not be discussed in detail here since their development and input-dependent circuit integration have recently been reviewed^15^

**Figure 3:**
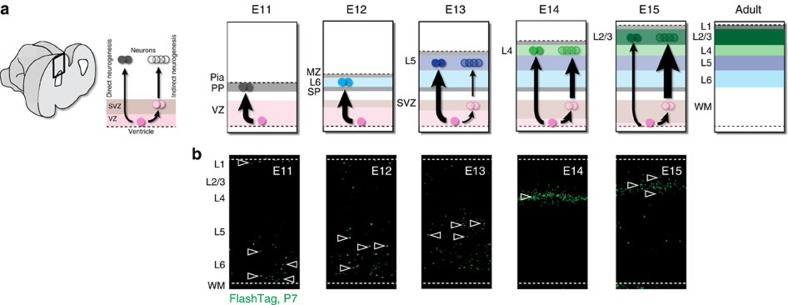
Direct and indirect neurogenic pathways during corticogenesis. (**a**) The neocortex is built in an inside-out manner in which neurons born from deeply located germinal zone migrate past earlier-born neurons to reside in more superficial layers. Note that initially, the preplate (PP) is split into a subplate (SP) and superficially located marginal zone (MZ) by incoming L6 neurons, such that early born neurons are later found in L1. Direct neurogenesis from the ventricular zone (VZ) predominates at early developmental stages, while indirect neurogenesis from the subventricular zone (SVZ) progressively increases during corticogenesis. E: Embryonic day; MZ: marginal zone; PP: preplate; SP: subplate. (**b**) FlashTag (FT) labelling highlights neurons born in the VZ through direct neurogenesis (ref. 55).

Glutamatergic neurons migrate radially into the cortex from the pallial VZ, which they populate in an inside-out manner (Fig. 3a). During early corticogenesis (until about E10.5 in mice), VZ progenitors initially self-amplify (at these initial stages, they are called neuroepithelial cells), and then begin giving rise directly to neurons (at this stage they are referred to as ‘radial glia’). As corticogenesis proceeds, ‘direct’ neurogenesis decreases; instead, VZ progenitors increasingly generate intermediate progenitors (transit amplifying cells, also called basal intermediate progenitors), which accumulate between the VZ and the developing cortical plate to form an additional dorsal germinal zone, the subventricular zone (SVZ)^30^,^31^ (Fig. 3). In contrast to what was previously thought, indirect neurogenesis is already present at early stages of neurogenesis (as evidenced by the presence of TBR2^+^ intermediate progenitors early in development (ref. 32)). Similarly, direct neurogenesis gradually tapers off into late developmental stages^33^. Thus supragranular L2/3 neurons are not exclusively born from intermediate progenitors, nor are infragranular neurons exclusively born from VZ progenitors. Compared to direct neurogenesis, indirect neurogenesis introduces a developmental shift (due to the additional round of cell division that takes place in the SVZ) such that neurons born indirectly are younger and less mature than their directly born counterparts. Within a single layer, excitatory neurons with distinct developmental trajectories and dates of birth thus coexist, and potentially display distinct developmental plasticities and circuit properties.

Cortical size depends on the net balance between amplifying divisions, which give rise to new progenitors, and differentiative divisions, which give rise to postmitotic neurons^34^,^35^. Indirect neurogenesis increases the final number of neurons by amplifying the progenitor pool. This is thought to be a critical step in gyrification, the process through which the neocortex becomes folded in some mammals. This process allows an increase in cortical surface and neuron number within the confined volume of the cranium. The increase in cortical size is particularly striking in supragranular layers (that is, L2/3), suggesting that cortico-cortical connections increase disproportionately compared to subcortical connections in gyrencephalic species.

Indirect neurogenesis and a corresponding SVZ is not, however, a hallmark of gyrification: it is present in lissencephalic mammals (for example, the mouse) and is found in other regions of the brain (for example, there is indirect neurogenesis in the ventral pallium, including in the thalamus^36^). One structure, however, the outer subventricular zone is conspicuously present in primates, but not other species, and contains multiple progenitor cell types thought to play a prominent role in cortical expansion^30^. Intriguingly, recent evidence suggests that mechanical factors could be sufficient to account for differences in gyrification across species, without the need for region-specific proliferation^37^. Finally, input-dependent controls over progenitor proliferation via thalamocortical afferents have been reported, which could in principle contribute to area-specific differences in cytoarchitectures and cell types^38^,^39^,^40^.

Additional means of generating specialized neuronal cell types include the presence of distinct neuronal progenitor types within the VZ. Morphologically and genetically distinct progenitor subtypes have indeed been reported (for example, short neuronal precursors, also called apical intermediate progenitors), but whether they relate to the generation of distinct neuronal cell types is still unclear^41^. An area of intense research is whether fate-restricted VZ progenitors exist (that is, progenitors which can only give rise to a subset of cortical neurons), or whether there is a single-progenitor type whose competence progresses throughout development^42^,^43^. This question has been difficult to investigate because it requires assessing the progeny of single progenitors with clonal resolution *in vivo*. In this context, progenitors can sequentially give rise to distinct molecularly defined neuronal cell types *in vitro*^44^,^45^, and classical transplantation experiments in ferrets suggest that progenitors can acquire the competence to generate normally later-born, but not earlier-born neurons^46^. Finally, at the tail end of corticogenesis, astrocytes are generated, seemingly from a subset of the progenitors that have formerly given rise to neurons^43^,^47^. Since astrocytes retain a proliferative potential, they represent a potentially interesting source to generate neurons through re-activation of developmental programs^48^.

The recently-discovered presence of DNA mosaicism in postmitotic neuron, perhaps resulting from DNA rearrangements immediately following mitosis^55^ represents an additional potential source of neuronal functional diversity. Such mosaicism may contribute to inter-individual differences in cell types, circuits and behaviour, and may be relevant to the broad spectrum of psychiatric disorders. If clinically relevant, diagnosis of such conditions will be challenging since causal mutations are only present in affected neurons and would not be detected by classical methods of DNA collection, such as buccal swabs or blood samples.

### Neocortical neuron specification and migration

Several transcription factors control the differentiation and function of specific neuronal subpopulations of cortical neurons^1^. These include FEZF2 (refs 50, 51) and CTIP2 (ref. 52) for L5B corticospinal neurons, and SATB2 for intracortical projection neurons^53^,^54^. While the initial events that control acquisition of neuron-type specific features following mitosis remain poorly characterized, we have recently shown that early neuronal differentiation is directed by a series of transcriptional waves whose sequence is critical for normal progression through development^55^. The transcriptional programs that drive neuronal identity are thus progressively implemented following mitosis. Supporting this finding, direct reprogramming of L4 (ref. 56) or L2/3 (ref. 57) neurons into L5B corticospinal neurons by overexpression of FEZF2 is more complete when performed early in neuronal development, suggesting a progressive decrease in plasticity. Thus, following mitosis, neuronal fate becomes progressively restricted rather than irreversibly fixed.

The cellular and molecular mechanisms controlling neuron migration from the VZ to the cortex have been well described, in particular with regard to the migration along radial glia processes and to the critical role of extracellular Reelin^58^. However, the cell-type specific processes controlling final laminar location remain poorly understood. From mid-corticogenesis on, there appears to be a tight relationship between date of birth and laminar position, since VZ-born isochronic neurons align along a narrow sublamina in L4 and L2/3, but at earlier stages the relationship between date of birth and precise laminar location seems less tightly coupled (Fig. 3b)^55^.

In addition to cell-intrinsic genetic programs and extracellular molecular gradients, activity-dependent processes also control neuronal migration. This has been well demonstrated for the tangential migration and differentiation of specific populations of GABAergic interneurons^59^. These cells can be recruited to specific target regions in an input-dependent manner, as shown both in the neocortex^60^ and in the thalamus^61^, providing an input-dependent mechanism for homeostatic regulation of circuit excitability. Compared with GABAergic interneurons, early stages of differentiation of glutamatergic excitatory neurons appear to be less dramatically affected by activity. Cortical lamination proceeds largely normally in the absence of vesicular neurotransmitter release^62^, or in the absence of input/output neocortical connectivity^63^, although the morphology and connectivity of neocortical neurons is likely to be affected. Supporting this possibility, chronic hyperpolarization of intracortical projection neurons and thalamic neurons affects axonal elongation and arborization^64^,^65^,^66^, and sensory input affects interhemispheric connectivity^67^.

### Self-organizing properties of neocortical circuits

During normal development, the laminar and tangential distribution of neocortical neurons is intimately linked with their ability to form circuits with one another. Location is thus a key determinant of competence of neurons to assemble into circuits, and disorders of cortical neuron migration may result in aberrant connectivities, perhaps reflecting loss of topographical guidance cues. Accordingly, neuronal migration disorders are often associated with epilepsy and intellectual disability in mice and humans, suggesting abnormal connectivities^68^. This may also apply to psychiatric disorders such as autism, which have been linked with migration defects in humans^69^, and common pathways may in fact link certain types of autism and epilepsy during development^70^,^71^. Although still little is known on the abnormal circuit features in autism spectrum disorders, early impairments in intermediate progenitors and migrating neurons has been reported in two mice models (22q11 deletion, Fragile X)^71^,^72^, as well as abnormal neuronal positioning in human patients^69^, suggesting that early occurring developmental events play a critical role in the behavioural phenotype.

**Figure 4:**
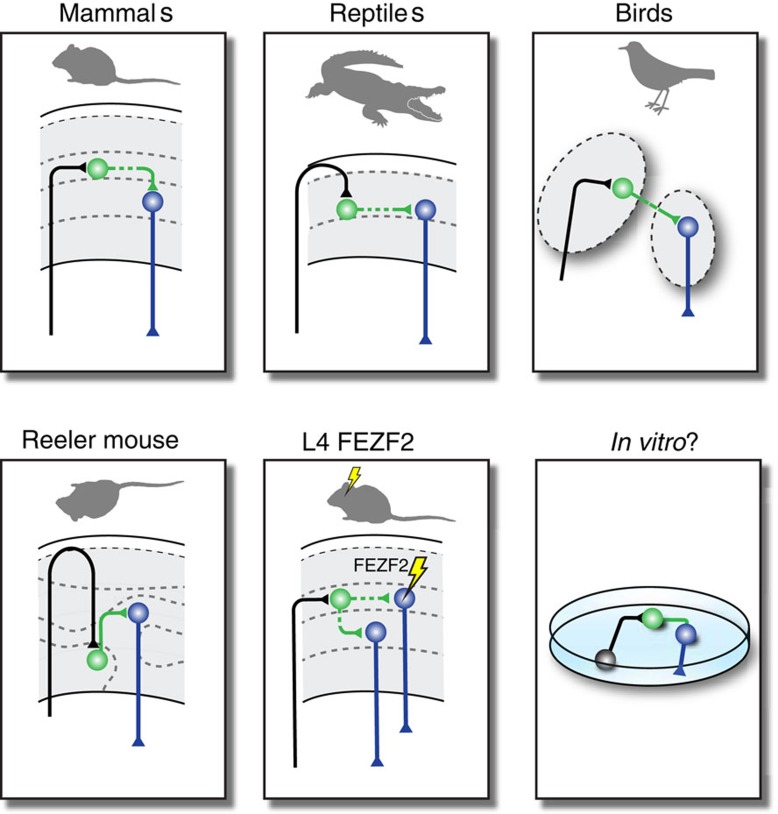
Self-organizing properties of neocortical neurons. The assembly of neurons into circuits may to some extent be independent of the spatial relationships between these neurons, due to the existence of conserved, location-independent molecular controls. Input neurons (in black) connect to intracortically projecting neurons (in green), which project to output neurons (in blue). A current limitation in testing this hypothesis is our generally limited ability to establish unambiguous correspondences in cell types across species/conditions. Reeler mouse, see ref. 74; L4 FEZF2 overexpression, see ref. 56.

On the other hand, key features of connectivity appear to be largely independent of neuronal location. Heterotopic FEZF2-expressing neurons still project to the spinal cord^50^ and are innervated by the appropriate complement of inhibitory interneurons^14^, and circuit connectivity is reprogrammed in a L5B-like fashion in L4 neurons which overexpress FEZF2^57^ (Fig. 4). Heterotopic L4 neurons appear to attract thalamocortical axons^73^, and in the Reeler mouse, key aspects of connectivity seem preserved despite a grossly abnormal cortical lamination^74^. The ability to form at least partially preserved circuits despite the anatomical shuffling of circuit elements is also present in other structures such as the zebrafish tectum, in which functional projections from the retina are preserved in mutants where tectal lamination is abnormal^75^. Thus, circuit assembly can also proceed to some extent independently of neuronal location. Comparative neuroanatomy also provides illustrations of this principle: Karten’s ‘equivalent circuit’ hypothesis^76^ postulates that corresponding circuits exist across species irrespective of their spatial layout (which includes whether the neurons that compose these circuits are organized in layers or nuclei) (Fig. 4). Although appealing, this hypothesis has been difficult to demonstrate formally because of the difficulty of defining strictly equivalent cell types independently of their position across species. The advent of single-cell RNA sequencing, offering the ability to establish correspondences across cell types based on shared transcriptional signatures may offer an opportunity to experimentally address this question.

Understanding the self-organizing properties of neuronal circuits is particularly important for regenerative and reparative medicine, including in the context of neuronal transplantations, since the reverse engineering of functional neuronal circuits may not require a precise replication of the topographical relationships which normally exist *in vivo*. Likewise, the recent development of organoid technologies, in which neuronal tissue is able to some extent to self-organize in three dimensions and potentially reproduce some of the core circuit features of the normal brain will be important to dissect out the input-dependent and cell-intrinsic features of circuits^77^.

### Emergence of topographic neuronal maps

Since the entry point of specific types of thalamic inputs into the cortex coincides with the presence of distinctive cytoarchitectural features (e.g. barrels in S1), a lot of effort has gone into understanding the cellular and molecular mechanisms through which input affects cortical neuron differentiation. This question has been particularly well studied in S1 and V1. Within these sensory areas, there is a topographical representation of the sensory periphery on the cortical surface, whereby neighbouring neurons respond to activation of neighbouring peripheral receptors, and where densely innervated regions occupy proportionally large regions of the cortical representation. This topographical layout is called somatotopy in S1, retinotopy in V1 and tonotopy in A1.

In S1, the input dependence of these maps was originally studied by lesioning sensory input pathways, such as by section of the infraorbital nerve, which conveys input from the whiskers, or by follicle cautery. These approaches consistently lead to impaired barrel pattering, with shrinkage/disappearance of injured whisker representations and expansion of remaining ones^12^,^78^. These results, however, cannot unambiguously be ascribed to purely developmental mechanisms, since injury-related processes such as axonal sprouting or neuronal death may be at play. To circumvent these limitations, pharmacological attempts have been made to manipulate sensory input, but dose-dependent effects and lack of specificity limit the interpretation of these studies. More recently, manipulations of neuronal activity with cell-subtype specificity, together with transcriptional analyses have enabled a better understanding of the molecular and cellular mechanisms that control the assembly of neuronal maps. These studies have shown that synaptic release of glutamate from the thalamocortical axons is required for the assembly of L4 neurons into barrels and dendritic polarization towards these axons, in particular *via* activation of NMDA receptors and metabotropic glutamate receptors^17^. In addition, several transcription factors, including Lhx2, Npas4, Zbtb20 function to polarize L4 dendrites towards incoming VB axons in S1 (refs 79, 80, 81), and Btbd3 has a similar role in V1 (ref. 82).

In the absence of exteroceptive, first-order thalamic input, primary areas (S1, V1) acquire molecular properties of associative cortical areas, such as S2 and V2 (refs 79, 83, 84). Interestingly, in the case of the somatosensory cortex, deprived S1 circuits acquire S2-like features (that is, an increase in excitatory/inhibitory ratio), suggesting that thalamic input not only affects L4 neurons but also determines downstream circuit assembly^79^. These findings suggest that associative cortical identity is a ground-state feature, and that acquisition of primary cortical area circuit properties is imparted by first-order thalamic input.

A similar process is at play within sensory thalamic nuclei, where input ablation experiments support the idea that higher-order genetic identity is a default feature, and that first-order identity is acquired in an input-dependent manner^19^. For example, in the absence of retinal input, the exteroceptive visual nucleus LGN receives input from L5B (refs 19, 85), a normally higher-order nucleus-destined afferent, and develops a corresponding higher-order transcriptional identity^19^. Ascending exteroceptive and descending corticofugal inputs may thus compete to innervate thalamic nuclei.

From an evolutionary perspective, the findings above support the view that neurons in primary areas and first-order nuclei may have emerged from ancestral secondary/higher-order-type neurons^86^,^87^. First-order neurons may have been co-opted from a ground-state pool of higher-order type neurons based on their ability to convey signals generated by high-resolution body receptors because of specific metabolic, electrophysiological and connectivity features^19^.

### Permissive versus instructive role of activity

The role of spontaneous activity in circuit assembly has been particularly well studied in the visual cortex, where emergence of binocular vision depends on the existence of ‘retinal waves’, which encode spatial proximity in temporal terms (that is, cells that are close to one another in the retina depolarize successively)^88^. These waves reach target visual structures in the central nervous system, including the visual cortex^89^, where they act to organize visual circuits. Retina-derived signals generated during vision are critical for the transcriptional activity and circuit assembly of visual cortical neurons^90^, but the precise molecular mechanisms at play are still poorly understood. Seemingly spontaneous wave-like patterns of activity have also been reported across the developing neocortex (bioRxiv 012237; doi: https://doi.org/10.1101/012237) and in the developing thalamus^91^ where in the latter case they appear to control the size of target cortical areas before sensory experience.

A recurring question in the study of input-dependent differentiation of neurons and circuits is whether input signals plays a permissive or instructive role on their postsynaptic targets. Does input activity act as a trigger for cell-intrinsic differentiation programs (permissive), or is a specific pattern of activity required to induce and sustain corresponding differentiation programs (instructive)? For example, following loss of VB input, acquisition of Po input by L4 neurons in S1 is not sufficient to rescue their differentiation; VB input thus appears to be instructive for these cells^79^. Modern optogenetic techniques now enable the distinction between instructive and permissive signals by allowing manipulations of the pattern of input activity, while keeping the overall charge of activity constant. Using such approaches, temporal patterns of binocular retinal activity have been shown to instruct eye-specific segregation in the developing visual system^92^. The molecular mechanisms through which distinct subtypes of neurons respond to such activity are poorly understood. Immediate early genes, such as c-fos and CREB, and Ca^2+^-dependent signals have been shown to play critical roles and have been studied in detail^93^, but how they relate to specific circuit properties and cellular identities is still largely unknown. Recently, however, the activity-dependent transcription factor NPAS4 has been shown to increase inhibition onto excitatory neurons specifically^15^,^94^, suggesting that a systematic cell-type specific dissection of input-dependent molecular pathways will be critical to fully understand the role of input on circuit assembly.

### Cortex-intrinsic and cortex-extrinsic development processes

In addition to the input/activity-dependent processes described above, cortex-intrinsic determinants of areal identity are also at play^95^. For example, COUP-TF1 is expressed in a rostral-low to caudal-high gradient and is necessary for the emergence of sensory areas, which are atrophied and shifted posteriorly in loss-of-function mice^96^. Similarly, ectopic FGF8 is necessary and sufficient to induce an S1-like area during development^97^. The cellular and circuit mechanisms that give rise to these macroscopic cytoarchitectural features are generally poorly understood, but likely involve effects both on progenitors and postmitotic neurons^98^.

The role of cortical area location on neuronal differentiation has been studied using transplantation studies, but has yielded conflicting results. For example, late embryonic rat visual cortex transplanted to neonatal primary somatosensory cortex develops barrel-like structures^99^, suggesting that cortical neurons can be respecified by somatosensory afferents, although this has not been examined with cellular or molecular resolution. In contrast, in a more recent study, embryonic stem cell-derived neurons transplanted into the motor cortex expressed molecular markers and connectivities typical of visual cortex neurons, and survival required a match between the areal identity of grafted neurons and transplantation site^44^,^100^. Thus, the plasticity of molecular differentiation programs in response to area-specific environmental/input-dependent cues may depend on cell-type- and developmental stage-specific factors of the grafted cells and their host tissue.

## Outlook

The level of coordination required for the assembly of distinct subtypes of neurons into specialized functional circuits across space and time is staggering, and raises a number of questions. What is the level of cellular diversity necessary to sustain the functions of the neocortex, and which are the features that delineate these core cell types? How do these features emerge during development and how do they vary across individuals, or in interaction with the environment? To which extent are they involved in the emergence of neurodevelopmental and neuropsychiatric disorders? Studies involving ‘non-clonal’ model animals might contribute to better define the normal spectrum of variability in cell positioning and circuit assembly, and raising animals in more natural environments could be used to gauge the impact of experience of this process. While these protocols will introduce natural ‘noise’ in the system, the increase in the resolution of the tools we use to manipulate and assess neurons and circuits, including single-cell RNA sequencing, single-cell optogenetics and targeted gene editing, will contribute to refine the readout of these studies and provide a more truthful picture of the degrees of freedom in cortical assembly, and on the limits between normal and abnormal development.

Understanding the number and nature of the independent parameters that define the configuration of the neocortex will be critical in attempts to reverse engineer developmental processes. Identifying these parameters will be important not only to define the relationship between developmental gene expression and mature neuronal function, but also to account for inter-individual variability in brain circuits and behaviour in both normal and pathological settings.

## Additional information

**How to cite this article:** Jabaudon, D. Fate and freedom in developing neocortical circuits. *Nat. Commun.* **8,** 16042 doi: 10.1038/ncomms16042 (2017).

**Publisher’s note:** Springer Nature remains neutral with regard to jurisdictional claims in published maps and institutional affiliations.

## Supplementary Material

Peer Review File
