# Supplementary material for: Fate and freedom in developing neocortical circuits
Source: Nat Commun. 2017 Jul 3;8:16042. doi: 10.1038/ncomms16042 (PMC5500875; doi:10.1038/ncomms16042)
Supplement: Peer Review File [file ncomms16042-s1.pdf]

Type of file: PDF
Size of file: 0 KB
Title of file for HTML: Peer Review File
Description:
